# Supplementary material for: The interactome of intact mitochondria by cross-linking mass spectrometry provides evidence for coexisting respiratory supercomplexes
Source: Mol Cell Proteomics. 2017 Dec 8;17(2):216–32. doi: 10.1074/mcp.RA117.000470 (PMC5795388; doi:10.1074/mcp.RA117.000470)
Supplement: Supplemental Data [file supp_RA117.000470_133922_0_supp_23978_fzffwf.zip › spectra_annotation/mito_spectra_annotation/309-1-18-1-11-1.pdf]

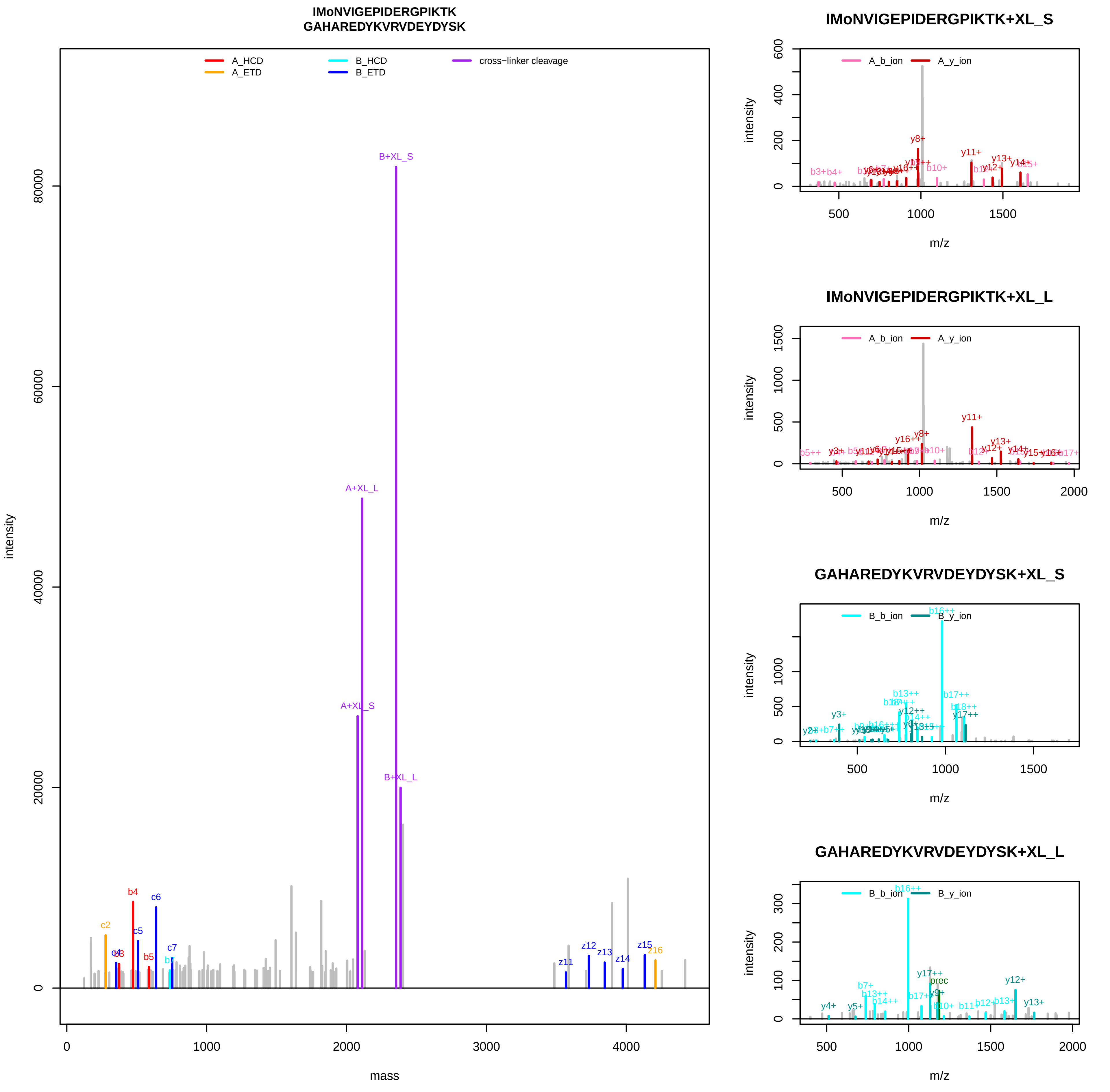
IMoNVIGEPIDERGPIKTK
GAHAREDYKVRVDEYDYSK
A_HCD
A_ETD
B_HCD
B_ETD
cross−linker cleavage
B+XL_S
A+XL_L
A+XL_S
B+XL_L
c2
c4
b3
b4
c5
b5
c6
c7
z11
z12
z13
z14
z15
z16
intensity
mass
IMoNVIGEPIDERGPIKTK+XL_S
IMoNVIGEPIDERGPIKTK+XL_L
GAHAREDYKVRVDEYDYSK+XL_S
GAHAREDYKVRVDEYDYSK+XL_L
A_b_ion
A_y_ion
B_b_ion
B_y_ion
y8+
y11+
y12+
y13+
y14+
b10+
y3+
y16++
b16++
b17++
b18++
b13++
y12++
y17++
y3+
y17++
prec
y9+
b7+
y4+
y5+
b11+
b12+
b13+
y12+
y13+
m/z
